# A Structural View on the Maturation of Lanthipeptides

**DOI:** 10.3389/fmicb.2020.01183

**Published:** 2020-06-09

**Authors:** Marcel Lagedroste, Jens Reiners, C. Vivien Knospe, Sander H. J. Smits, Lutz Schmitt

**Affiliations:** ^1^Institute of Biochemistry, Heinrich Heine University Düsseldorf, Düsseldorf, Germany; ^2^Center for Structural Studies, Heinrich Heine University Düsseldorf, Düsseldorf, Germany

**Keywords:** structural biology, biochemistry, lanthionine, enzymes, protein–protein interaction

## Abstract

Lanthipeptides are ribosomally synthesized and posttranslationally modified peptides, which display diverse bioactivities (e.g., antifungal, antimicrobial, and antiviral). One characteristic of these lanthipeptides is the presence of thioether bonds, which are termed (methyl-) lanthionine rings. These modifications are installed by corresponding modification enzymes in a two-step modality. First, serine and threonine residues are dehydrated followed by a subsequent catalyzed cyclization reaction, in which the dehydrated serine and threonine residues are undergoing a Michael-type addition with cysteine residues. The dedicated enzymes are encoded by one or two genes and the classification of lanthipeptides is pending on this. The modification steps form the basis of distinguishing the different classes of lanthipeptides and furthermore reflect also important mechanistic differences. Here, we will summarize recent insights into the mechanisms and the structures of the participating enzymes, focusing on the two core modification steps – dehydration and cyclization.

## Introduction

**FIGURE 1:**
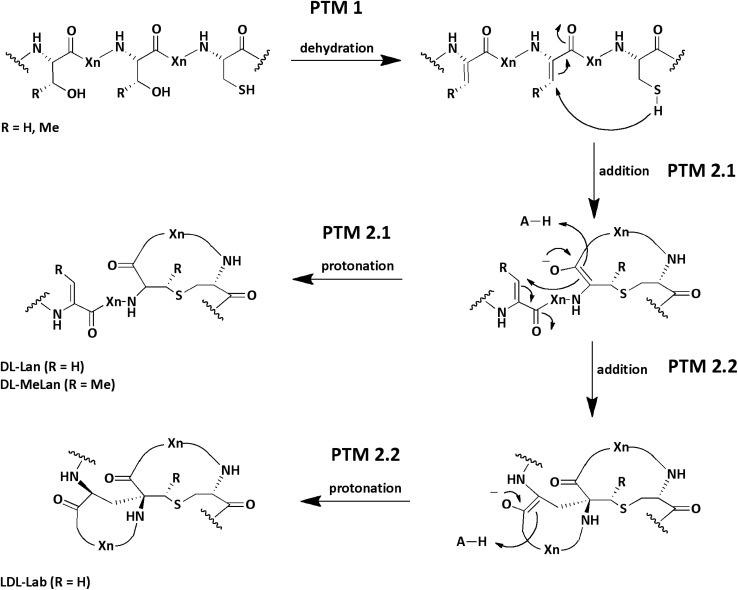
Reaction and structure of common PTMs in lanthipeptides. During a first modification step (PTM 1) a dehydratase catalyzes the dehydration of serine and threonine residues (Dha, Dhb). In a second step (PTM 2.1) a cyclase catalyzes the Michael-type addition of a cysteine residue to a dehydrated amino acid. Within the active center an acid (H-A, e.g., His) protonates the enolate. Finally, the Lan or MeLan rings are formed. In some lanthipeptides an additional Michael-type addition reaction (PTM 2.2) is catalyzed by the cyclase yielding a Lab amino acid. In the scheme the stereochemistry of the chiral center in the final products are exemplary (e.g., DL-Lan; but LL-Lan is also possible). The acronym X_n_ stands for n-quantity amino acids. The scheme is modified and based on (Repka et al., 2017).

Ribosomally synthesized and posttranslationally modified peptides (RiPPs) are a large family of natural compounds of diverse biological functions (Arnison et al., 2013). Among the RiPPs, lanthipeptides form the largest sub-family (Skinnider et al., 2016), which is characterized by the presence of multiple lanthionine (Lan) or (methyl-) lanthionine rings ((Me)Lan)-, that restrict the conformational flexibility of the peptides and give rise to their high biological stability (Bierbaum et al., 1996). Common to lanthipeptides is the ribosomal biosynthesis of a precursor peptide that is composed of an N-terminal leader peptide (LP) and a C-terminal core peptide (CP), termed LanA (Oman and van der Donk, 2010; Arnison et al., 2013). While all posttranslational modifications (PTMs) are introduced only in the CP, the LP increases the efficiency of the PTMs to the lanthipeptide by its PTM machinery and keeps the peptide in an inactive state prior to translocation (van der Meer et al., 1994; Kuipers et al., 1993, 2004; Khusainov and Kuipers, 2012). The fully modified lanthipeptide is termed mLanA (Arnison et al., 2013). Subsequently, the LP is proteolytically removed either before or after secretion to the extracellular space via its cognate ABC transporter and the active lanthipeptide is released into the extracellular space (van der Meer et al., 1993, 1994; Nishie et al., 2011; Ortega et al., 2014). In general, all lanthipeptides share at least two common PTMs. The first one is the dehydration of serine and threonine residues, resulting in the formation of 2,3-didehydroalanine (Dha from serine) and 2,3-didehydrobutyrine (Dhb from threonine) (Figure 1; Gross and Morell, 1967, 1971; Gross et al., 1969). This reaction is catalyzed by the dehydratase LanB or dehydratase domains depending on the classification of the lanthipeptide (see next section) (Gilmore et al., 1994; Gutowskieckel et al., 1994; Peschel et al., 1996; Karakas Sen et al., 1999). The second common PTM is the Michael-type addition of a cysteine side chain with the previously dehydrated amino acids yielding *meso*-lanthionine (from Dha) or (3-methyl-) lanthionine (from Dhb) (Figure 1) introduced by the cyclase LanC (Gross and Morell, 1967, 1971; Gross et al., 1969; Barber et al., 1988). Additionally to these two PTMs that are the foundation of lanthipeptides, a range of further modifications such as labionin (Lab) ring formation (Figure 1; Meindl et al., 2010; Iorio et al., 2014) or tailoring reactions such as halogenation of tryptophan residues, decarboxylation or acylation have been observed (Mortvedt et al., 1991; Kupke et al., 1992; Skaugen et al., 1994; van de Kamp et al., 1995; Heidrich et al., 1998; Ekkelenkamp et al., 2005; Castiglione et al., 2008; He et al., 2008; Velasquez et al., 2011; Huang and Yousef, 2015). However, these reactions are not further discussed in this review and the reader is referred to excellent reviews covering these aspects (Mortvedt et al., 1991; Kupke et al., 1992; Skaugen et al., 1994; van de Kamp et al., 1995; Heidrich et al., 1998; Ekkelenkamp et al., 2005; Castiglione et al., 2008; He et al., 2008; McIntosh et al., 2009; Meindl et al., 2010; Velasquez et al., 2011; Arnison et al., 2013; Dunbar and Mitchell, 2013; Iorio et al., 2014; Walsh, 2014; Huang and Yousef, 2015; Ortega and van der Donk, 2016; Repka et al., 2017).

**FIGURE 2:**
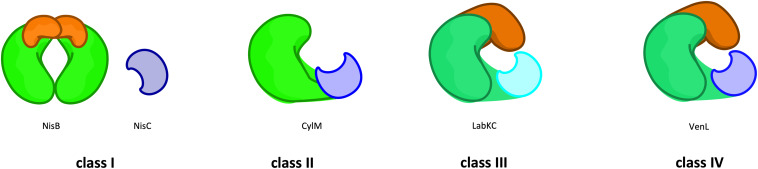
Classes of lanthipeptides based on the modification machineries. The classification of lanthipeptides depends on their modification enzymes and their domain organization as well as those PTM mechanisms. In class I (e.g., NisB) the first PTM (dehydration) is catalyzed by a glutamylation/elimination-domain (green/orange). The dehydration in class II (e.g., CylM) take place in a kinase-domain (green). In class III (LabKC) and IV (VenL) this modification is catalyzed by a lyase/kinase-domain (dark green/dark orange). The second PTM (cyclization) is catalyzed by the zinc-dependent LanC enzyme (e.g., NisC; blue) or LanC-like domain (light blue), whereas in class III the LanC-like domain is independent from a zinc-ion (cyan). For further details see text.

In 2013, a new nomenclature was suggested that subdivides lanthipeptides based on their modification machinery in four families, termed class I–IV (Figure 2; Arnison et al., 2013) that are described in greater detail in the following sections. In this review, we will follow this new nomenclature. Furthermore, we will restrict ourselves to the two common maturation steps that occur in the cytosol of lanthipeptide producing strains.

## Lantibiotics – Specialized Lanthipeptides

The hallmark of lanthipeptides is the presence lanthionine or (methyl-) lanthionine rings. In cases that lanthipeptides possess antimicrobial activity they are called lantibiotics (Schnell et al., 1988; Arnison et al., 2013). However, other activities, such as antifungal, antiviral, morphogenetic, or antinociceptive have been described (Kodani et al., 2004, 2005; Ferir et al., 2013; Iorio et al., 2014; Mohr et al., 2015). The antimicrobial activity, which is mainly directed against Gram-positive bacteria where the target of most lantibiotics is the membrane and/or a specific receptor. A prominent example, nisin, a lantibiotic produced by *Lactococcus lactis*, targets the peptidoglycan precursor lipid II. Nisin contains five (Me)Lan rings, where the first two bind to the pyrophosphate moiety of lipid II and directly inhibit the cell wall synthesis. Additionally, nisin and lipid II molecules form pores in the cell membrane of the target cell in a stoichiometry of eight nisin and four lipid II molecules (Severina et al., 1998; Breukink et al., 1999; Wiedemann et al., 2001; Brumfitt et al., 2002; van Heusden et al., 2002; Hasper et al., 2004, 2006; Hsu et al., 2004; Chatterjee et al., 2005b; Breukink and de Kruijff, 2006; Lubelski et al., 2008; Bierbaum and Sahl, 2009; Schneider and Sahl, 2010; Islam et al., 2012). Despite its usage in the food industry for almost 50 years (Cotter et al., 2005) this dual mode of action explains why hardly any acquired resistances have been described in the literature.

## The First Maturation Step – The Dehydration Reaction

The major discriminators among the four classes of lanthipeptides are the lanthipeptide modification enzymes. Here, four different routes corresponding to the four subfamilies (LanB, LanM, LanKC, and LanL) have evolved, which mainly differ in the mechanism of dehydrating serine and threonine residues (Figure 2).

**FIGURE 3:**
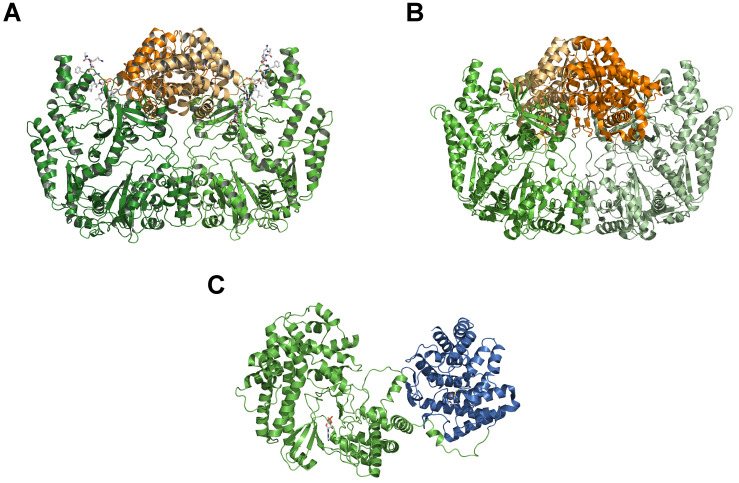
Comparison of LanB and LanM crystal structures. **(A)** Crystal structure of dimeric NisB (PDB ID: 4WD; Ortega et al., 2015), representing the first example of a class I LanB dehydratase. The glutamylation domain is highlighted in green and light green, the elimination domain in orange and light orange. The part of the LP of NisA is shown in ball-and-stick representation. **(B)** Crystal structure of dimeric MibB (PBD ID: 5EHK; Ortega et al., 2016). Color-coding is as in panel **(A)**. **(C)** Crystal structure of CylM (PBD ID 5DZT; Dong et al., 2015), the first example of a LanM enzyme of class II lanthipeptides. The kinase-domain is shown in green and the cyclase-domain in blue. A bond AMP molecule within the kinase-domain is displayed as a ball-and-stick representation. Cartoons were generated using PyMol (www.pymol.org).

Class I family dehydratases (LanB) contain the well-studied enzymes NisB or SpaB that dehydrate their substrates nisin (NisA) or subtilin (SpaA), respectively (Gross and Morell, 1967; Gross et al., 1969). NisB adopts a dimeric state in solution and interestingly interacts with the different maturation states of NisA and not only with its cognate substrate (unmodified NisA) in the low micromolar range (Mavaro et al., 2011). *In vivo* co-expression studies of NisB and the cyclase NisC without purification resulted in dehydration and cyclization of NisA indicating functional enzymes (Karakas Sen et al., 1999; Kluskens et al., 2005; Rink et al., 2005; Rink et al., 2007a). In 2013, Garg et al. (2013) demonstrated *in vitro* activity of purified NisB by using extracts of *Escherichia coli* and subsequently identified the cytosolic extract to be the key element for glutamylation of NisA. Finally, the addition of glutamyl-tRNA (tRNA^Glu^), derived from glutamyl-tRNA synthetase, and glutamate to purified recombinant NisB restored the *in vitro* activity and consequently, polyglutamylated intermediates were identified by MS analysis (Ortega et al., 2015). This highlighted that the hydroxyl groups of serine and threonine in the CP of NisA were esterified with the alpha-carboxyl group of glutamate, where a cognate tRNA is the glutamyl-donor. Subsequently, the elimination of these activated residues resulted in the dehydrated residues Dha and Dhb. Of course, this reconstitution allowed a detailed study of the catalytic activity and the identification of essential amino acids of this LanB dehydratase (Garg et al., 2013; Bothwell et al., 2019). In 2015, the fruitful collaboration of the Wilfred van der Donk and Satish K. Nair groups also reported the crystal structure of NisB (Figure 3A). Similar to *in vitro* observations, NisB crystallized as a dimer (Ortega et al., 2015; Reiners et al., 2017). Importantly, NisB was co-expressed with NisA and parts of the LP including the FNLD box, which is pivotal for the interaction with NisB, were visible in the final electron density (shown in ball-and-stick representation in Figure 3A). In the crystal structure, the LP interacts with a twisted ß-strand resulting in an antiparallel, four-stranded ß-sheet. This resulted in a 2:2 stoichiometry of NisB:NisA, which is in contradiction to the *in vitro* data, which determined a 2:1 ratio of NisB:NisA using surface plasmon resonance (Mavaro et al., 2011). In 2019, a co-crystallization approach with a non-reactive substrate mimic also revealed a 2:2 stoichiometry of NisB-Val169Cys:NisA-Ser3Dap^Glu^-Ser(-12)Cys (Bothwell et al., 2019). Thus, the reason for this difference is still an open question.

NisB can be sub-divided into an N-terminal glutamylation domain (green and light green cartoons in Figure 3A of approximately 800 amino acid residues) and a C-terminal elimination domain (orange and light orange cartoons in Figure 3A of approximately 350 amino acid residues). This two-domain structural architecture was also found within MibB (Figure 3B), the class I LanB enzyme of NAI-107 (Ortega et al., 2016). Equally important, MibB like NisB requires tRNA^Glu^ to catalyze the dehydration reaction. Three further examples of related LanB enzymes use so-called split LanB, where one protein is involved in aminoacylation and the other protein in the elimination of activated amino acid (aa) residues (Hudson et al., 2015; Mohr et al., 2015; Ozaki et al., 2016), also depend on the presence of tRNA^Glu^ for dehydration. This clearly demonstrates that class I LanB enzymes use this rather unexpected mechanism to dehydrate serine and threonine residues in the CP of lanthipeptides.

**FIGURE 4:**
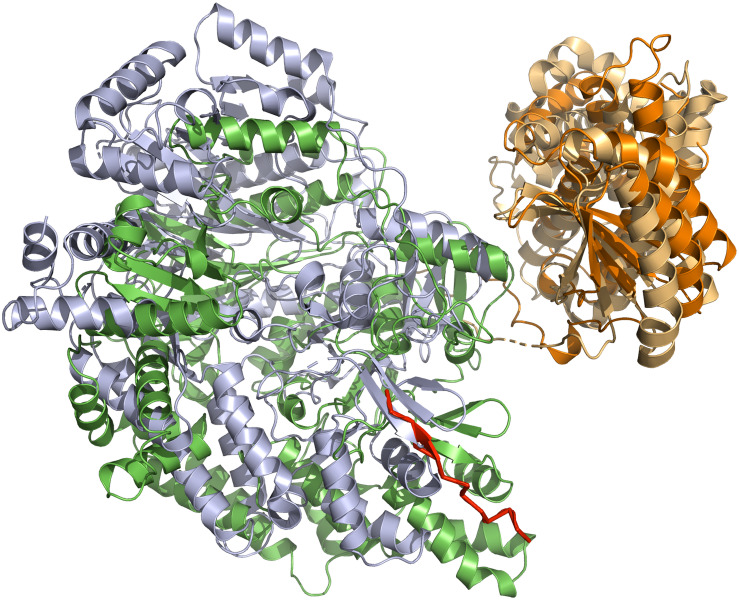
Superimposition of NisB and MibB crystal structures. The superimposition is based on the elimination domain (residues 713–961 of NisB and 800–1048 for MibB with a RSMD of 3.6 Å over 618 Ca atoms). The glutamylation domain is shown in green (NisB) and gray (MibB), while the elimination domain is shown in light orange (NisB) and orange (MibB). For simplicity only monomers of NisB and MibB are shown. The bound LP of NisA in NisB is shown in red. Cartoons were generated using PyMol (www.pymol.org).

In contrast to NisB (Ortega et al., 2015), MibB (Ortega et al., 2016) was crystallized in the absence of a substrate and displayed the same overall dimeric architecture composed of an approximately 800 amino acids large N-terminal glutamylation domain (green and light green in Figure 3B) and an approximately 350 amino acid large C-terminal elimination domain (orange and light orange in Figure 3B). The absence of the natural substrate allows the comparison with NisB to highlight structural changes that occur concomitant with substrate binding (Figure 4). As evident from the structural superimposition of both proteins using the C-terminal elimination domain as an anchor point, the glutamylation domain undergoes a translational and rotational motion resulting in a more compact shape of the LanB enzyme (Figure 4). This transition might be reminiscent of the conformational selection proposed for class II LanM enzymes. Here, the LanM enzyme is in equilibrium between an inactive and an active conformation. In the absence of substrate, more precise the LP, the equilibrium is shifted toward the inactive state, while binding of the LP shifts it toward the catalysis-competent state. This model is supported by experiments, in which the LP was added *in trans* or fused to the LanM enzyme (Levengood et al., 2007; Oman et al., 2012; Thibodeaux et al., 2015). In both cases, the isolated CP was modified although the fusion of the LP resulted in a more efficient system. Khusainov and Kuipers performed *in vivo* studies with separately expressed LP (NisA (1-23) and CP of nisin (NisA(24-57)-H_6_), leaderless nisin (NisA(24-57)-H_6_), and full-size nisin with a C-terminal extension and a His-tag (NisA(1-57)-H_6_). These studies revealed partially modifications in spite of missing or in *trans* expressed LP, which led to the conclusion that the LP is not crucial for PTMs but increases the efficiency. However, only the fused LP led to complete modification (Khusainov and Kuipers, 2012). All in all, a mostly similar scenario could be suggested for class I LanB enzymes. In contrast, the LP of class III lanthipeptides seems to be crucial for PTMs (Müller et al., 2011; Wang and van der Donk, 2012).

Structural information is available for class II LanM enzymes (Siezen et al., 1996; Zhang et al., 2012) that encode the dehydration, elimination and cyclization domains on a single gene (Figure 3C). In clear contrast to LanB enzymes, these dehydratases require ATP and Mg^2+^ as cofactors as shown experimentally for LctM in 2005 (Chatterjee et al., 2005a). The crystal structure of CylM (Dong et al., 2015) revealed the expected two-domain organization, an N-terminal dehydration domain (green in Figure 3C) and a C-terminal cyclization domain (blue in Figure 3C), which resemble the structure of NisC (Li et al., 2006), a class I LanC enzyme (see below). Not anticipated, the N-terminal domain displays structural similarities to eukaryotic lipid kinase domains bearing a novel, secondary structure topology. The activation loops of serine/threonine kinases including the P-loop are present as well as characteristic helices. Nevertheless, also a novel kinase activation domain is present, whose function was determined by mutational studies, explaining the dependence of LanM enzymes on ATP and Mg^2+^. Here, in contrast to LanB enzymes (Garg et al., 2013), the dehydration relies on phosphorylation of serine and threonine residues of the substrate, the presence of phosphorylated instead of glutamylated intermediates and subsequent the elimination of inorganic phosphate (Chatterjee et al., 2005a). However, only AMP was observed in the structure and conclusions on the molecular mechanism of LanM function are not available at the moment.

The only recently discovered Class III (LanKC) and class IV (LanL) lanthipeptide modification enzymes, display a three-domain organization composed of a lyase, kinase and a C-terminal cyclase domain, which differs among the two classes (Figure 2; Goto et al., 2010; Meindl et al., 2010; Zhang et al., 2015). While the cyclase domain of LanL is apparently similar to the C-terminal domain of LanM enzymes or LanC and its activity also clearly relies on Zn^2+^. On the contrary, the cyclase domain of LanKC is apparently not Zn^2+^-dependent as it does not contain the highly conserved residues that are required for the coordination of this ion. Thus, two classes depending on the ability to coordinate Zn^2+^ or not were defined and the generic names LanKC (Class III) or LanL (class IV) were introduced (Kodani et al., 2004; Goto et al., 2010). Structural information is so far not available and insights into these lanthipeptide modification enzymes depend solely on genetic and functional data. Sequence analysis revealed similarities to serine/threonine kinases and effector proteins from Gram-negative and Gram-positive bacteria that catalyze the elimination of phosphorylated serine and threonine residues (phospholyases) in the N-terminal part of the protein (Young et al., 2003; Zhu et al., 2007; Chen et al., 2008). In contrast to class II LanM enzymes that are strictly ATP dependent, the kinase domains of LanKC and LanL have no real specificity for a phospho-donor. Depending on the enzyme under investigation, specificities for GTP/dGTP, ATP, ATP/GTP/CTP/TTP or any NTP/dNTP were discovered (Muller et al., 2010; Krawczyk et al., 2012b; Voller et al., 2012; Wang and van der Donk, 2012; Jungmann et al., 2016). However, based on the sequence similarities of the lyase and kinase domains, it can be assumed that the mechanisms of phosphorylation and elimination are shared between LanKC and LanL enzymes.

We have now a fairly detailed understanding of how the dehydration reactions are catalyzed in the different classes of lanthipeptides synthetases. Nevertheless, class I enzymes represent a special case as the dehydratase LanB and the cyclase LanC are separately expressed enzymes that can act on their own (Kluskens et al., 2005; Li et al., 2006; Li and van der Donk, 2007; Rink et al., 2007a; Garg et al., 2013). However, *in vivo* both enzymes are present. An elegant set of experiments using plasmid-based expression of the possible combinations of maturation enzymes demonstrated an astonishing inter-dependence between the dehydratase NisB and the cyclase NisC. Here, ring formation and dehydration acted in concert, which resulted in the protection of potential dehydration positions during ring formation (Karakas Sen et al., 1999; Koponen et al., 2002; Kluskens et al., 2005; Li et al., 2006; Li and van der Donk, 2007; Rink et al., 2007a, b; Lubelski et al., 2009; Oman and van der Donk, 2010; Plat et al., 2011, 2013; Khusainov and Kuipers, 2012; Khusainov et al., 2013; Garg et al., 2013; Ortega et al., 2015). This resulted in the proposal, that a strict N- to C-terminal directionality is operational in NisA maturation, suggesting that dehydration and ring formation is an intertwined process (Ortega et al., 2015). Consequently, such a directionality would also suggested a sort of channeling of the substrate that is bound to a LanB/LanC complex forcing the PTM reactions to start at the N-terminus and proceed all the way to the C-terminus before finalizing the maturation reactions. In 2014, Zhang et al. (2014) confirmed for NisB by mass-spectrometry (more precisely via HSEE analysis) an overall dehydration process from N- to- C- terminus, but a closer view revealed no strict directionality. In clear contrast, *in vitro* studies of the lanthipeptide NAI-107 (MibA, class I), suggest the absence of a N- to C-directionality, rather a C- to N-directionality, after dehydration of the N-terminus was observed (Ortega et al., 2016). The same C- to N-directionality was found for class III LanKC enzymes via single-mutation-studies (AciKC) and isotope labeling studies (LabKC) (Krawczyk et al., 2012a; Wang and van der Donk, 2012). Contrary to this, the synthetases of class II LctM and HalM2 revealed the opposite modification direction from N- to C-terminus (Lee et al., 2009). Surprisingly, the directionality of ProcM, also a class II synthetase, is distinct from the previous mentioned LacM enzymes. Zhang et al. (2012) investigated beside NisB also the dehydration directionality of ProcM, which revealed a generally C- to N-terminal direction of the dehydration process via mass-spectrometry. The difference regarding the directionality of the modification process within one class (i.e., ProcM and HalM2) may indicate that different binding modes are present. ProcM and HalM2 are not phylogenetically closely related, which could have led to distinct binding modes for the lanthipeptides (Zhang et al., 2012). This obviously raises the question whether a unique mechanism is operational and what the molecular ruler underlying these mechanisms actually is. Further structural studies of synthetases (LanM, LanKC, and LanL) with the lanthipeptide LanA or LP are undoubtly necessary to clear up the remaining questions before conclusions can be drawn. All in all, the directionality of the dehydration process is non-uniform among the class I dehydratases (LanBs) and class II synthetases. Consequential, the directionality can not be assumed based on the class of the lanthipeptide and each modification enzyme needs to be investigated.

## The Second Maturation Step – The Cyclization Reaction

LanC enzymes and the cyclization domain of classes II–IV enzymes catalyze the nucleophilic attack of a thiolate (from Cys) to dehydrated amino acid (aa), where they facilitating the regio- and stereoselectivity to form thioether rings with the correct ring topology. Although, the lanthionine ring formation can occur spontaneously at basic pH values (pH > 7.5), however, leading to an erroneous stereochemistry of the Lan or (Me)Lan rings (Burrage et al., 2000; Okeley et al., 2000; Kuipers et al., 2004). Due to missing stereochemistry investigations of Lan and MeLan residues of lanthipeptides and the assumption that all Lan and (Me)Lan rings in lanthipeptides have the same “DL”-stereochemistry as previously shown for selected lantibiotics (Chatterjee et al., 2005b), the discovery that CylM can catalyze different stereochemistry within one single polypeptide was surprising. Furthermore, the studies of Tang and van der Donk (2013) revealed that the sequence of the lanthipeptide could be crucial for the stereoselectivity of ring formation.

**FIGURE 5:**
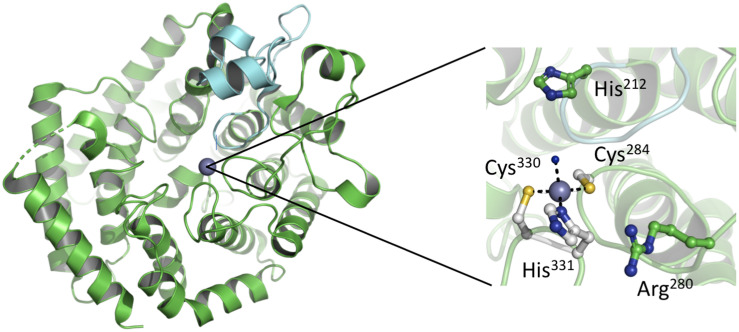
Crystal structure of NisC. **(Left)** The overall crystal structure comprises an a, a toroidal fold (green) and a domain extension. This extension a SH2-like domain (cyan) is located close to the catalytic center. Within the active site a Zn^2+^ ion, shown as a gray sphere, is bound. **(Right)** Zoom-in into the active site of NisC. Residues important for the coordination of the Zn^2+^ ion (Cys284, His330, and Cys331) or function (His212 and Arg280) are shown in ball-and-stick representation. Cartoons were generated using PyMol (www.pymol.org).

**FIGURE 6:**
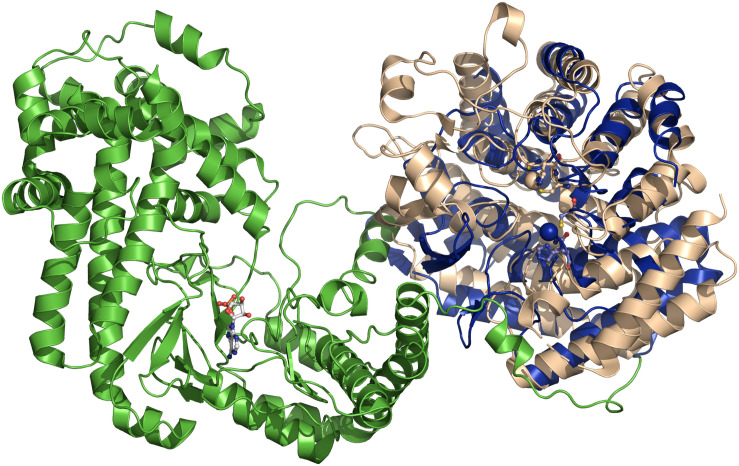
Superimposition of NisC and CylM crystal structures. The superimposition is based on the cyclase domain of CylM (residues 641–992 of CylM). NisC is shown in blue and CylM in green (dehydratase domain) and light brown (cyclase domain). The bound AMP of CylM is shown in ball-and-stick representation. The two Zn^2+^ ions and the coordination amino acids are shown as blue sphere (NisC) and light brown sphere (CylM). The coordination amino acid residues are highlighted in ball-and-stick representation in blue (NisC) and light brown (CylM). Cartoons were generated using PyMol (www.pymol.org).

The analysis of purified LanC enzymes (NisC and SpaC) revealed the presence of equal stoichiometric amounts of Zn^2+^ suggesting that the metal ion plays an essential role in deprotonating the thiol group of the cysteine residue, presumably by decreasing the pK_a_ value of the cysteine side chain (Okeley et al., 2003). Such a deprotonation or at least polarization accelerates the rate of Michael-type additions during the formation of the (Me)Lan rings. A detailed glimpse on the mechanism was possible, as the crystal structure of NisC was reported in 2006 (Li et al., 2006). The protein displays an a, a toroid consisting of six helices each, a SH2-like domain and one Zn^2+^ ion (Figure 5). The Zn^2+^ ion is coordinated by two highly conserved cysteine residues (Cys284 and Cys330) and one histidine residue (His331). Additionally, the tetragonal coordination sphere is complemented by a water molecule (inset of Figure 5). The presence of the SH2-like domain suggests that this domain interacts with dehydrated NisA, however there are no experimental evidence supporting this hypothesis and the functional role of this domain remains elusive. Based on the crystal structure of NisC, some residues conserved among LanC proteins were mutated (Li and van der Donk, 2007). There, the mutations of Cys284, Cys330, and His331 lead to inactive NisA. Interestingly, the ability to bind Zn^2+^ was preserved by mutating other active site residues (e.g., mutation H212N, H212F, and D141N) but no cyclization was detected. Thus, we slowly obtain a mechanistic picture of how NisC, but also LanC proteins in general, guide the formation of lanthionine rings. These insights will likely also hold for the cyclization domains of class II and class IV enzymes due to the structural conservation [Figure 6 – comparison of CylM (LanM) and NisC (LanC)] or the conservation of residues identified to be essential for Zn^2+^ coordination or enzyme function. Noteworthy, an SH2-like domain (Mayer and Gupta, 1998; Li et al., 2006), which is found in NisC and might be involved in substrate binding, is not present in CylM. There, an additional subdomain is included in the cyclization domain, that seem to be important for substrate binding (Dong et al., 2015). Nevertheless, we still do not have structural information of the LanA–LanC complex and only this information will result in a final and complete picture. In contrast, the putative cyclization domain of class III enzymes (LanKC, i.e., AciKC) do not contain the conserved Zn^2+^ coordinating residues, which raises the question how cyclization takes place (Wang and van der Donk, 2012). Another important aspect of class III lanthipeptides is the presence of another type of cyclization, Lab or methyllabionin (MeLab; Meindl et al., 2010; Iorio et al., 2014). After the initial Michael-type addition of a cysteine residue and Dha, the system undergoes another Michael-type addition reaction with a second Dha residue, resulting in the formation of a methylene moiety based ring, a (Me)Lab ring. However, one has to wait for structural insights into the LanKC family before further mechanistic conclusions can be drawn.

## A Concerted Action During Maturation

The individual domains of LanKC (class III) and LanL (class IV) enzymes are capable of catalyzing their individual reactions also in the absence of the other domains (Goto et al., 2011). This especially holds true for class I enzymes. However, early on, functional studies based on co-immunoprecipitation, yeast two hybrid approaches or mutational studies demonstrated that at least LanB and LanC act synergistically (Siegers et al., 1996; Kiesau et al., 1997; Lubelski et al., 2009). Moreover, a maturation complex consisting of not only NisB and NisC but also the ABC transporter NisT apparently exists during the modification of NisA (Siegers et al., 1996). Further support of a concerted action came from studies of subtilin, which suggested also the presence of such a complex composed of the dehydratase SpaB, the cyclase SpaC and the ABC transporter SpaT (Kiesau et al., 1997).

First insights into the assembly and architecture of a full class I lanthipeptide maturation complex was obtained for the lanthipeptide nisin. Reiners et al. (2017) used purified components to assemble the NisB/NisC/NisA maturation complex *in vitro*. Using size exclusion chromatography combined with multi-angle light scattering (SEC–MALS), they demonstrated that the complex was composed of a dimer of the dehydratase NisB, a monomer of the cyclase NisC and one molecule of the substrate, NisA resulting in a stoichiometry of 2:1:1 and a molecular weight of approximately 291 kDa. Importantly, the formation of the maturation complex was strictly dependent on the presence of the FNLD box within the LP as shown previously by *in vivo* and *in vitro* studies. Mutation of the four amino acids of the FNLD to AAAA completely prevented complex assembly (Khusainov et al., 2011, 2013; Mavaro et al., 2011; Plat et al., 2011, 2013; Abts et al., 2013). From a mechanistic point of view, it was also important that a molecular signal was identified in this study that triggered disassembly of the maturation complex. Using a series of (Me)Lan ring mutants, e.g., Cys–Ala exchanges that prevented ring formation at the corresponding position, proved that the presence of the last, C-terminal (Me)Lan ring represented the ‘disassembly signal’. This obviously goes nicely in hand with the *in vivo* situation, where a maturation complex should continue the PTM reaction and only release the fully modified product. In other words, the maturation complex is capable of reading out the stage of modifications and only the terminal modification state, the fully modified product is released, ready to be secreted by the cognate ABC transporter. Of course the exact molecular role of the ABC transporter within such a maturation complex is currently completely unknown and requires further investigations addressing its precise role.

**FIGURE 7:**
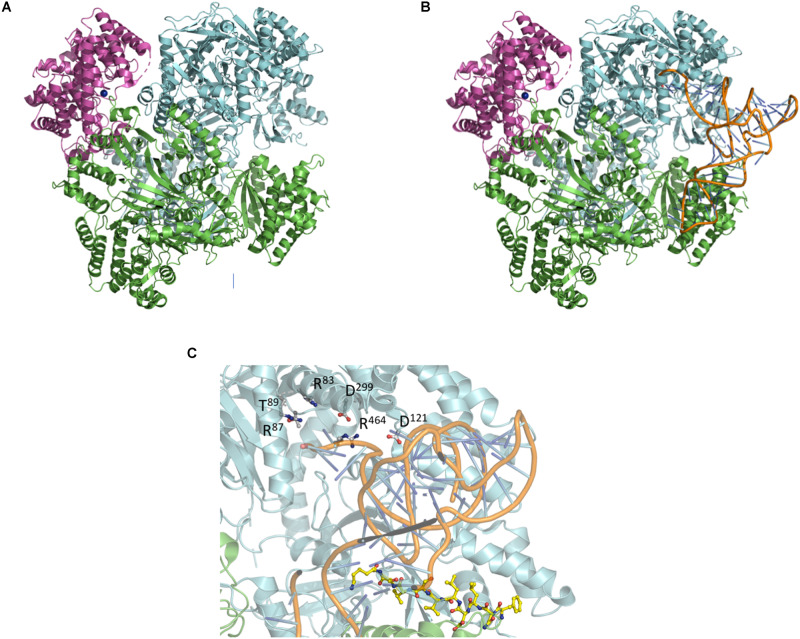
Composition of the NisA maturation complex NisB and NisC and tRNA docking. **(A)** Crystal structures of NisC and NisB docked into the SAXS envelope (Reiners et al., 2017). **(B)** Docking of tRNA^Glu^ into the NisA maturation complex composed of dimeric NisB (cartoon representation in green and cyan) and monomeric NisC (cartoon representation in magenta). The LP bound to NisB is shown in yellow ball-and-stick representation. **(C)** Zoom-in into the tip region of bound tRNA^Glu^. Residues of NisB (Garg et al., 2013; Khusainov et al., 2013; Ortega et al., 2015), which resulted in abolished dehydration upon mutation (Arg87, Thr89, Asp121, Asp299, and Arg464), are highlighted in ball-and-stick representation. Cartoons were generated using PyMol (http://www.pymol.org).

Moving one step further, small-angle X-ray scattering (SAXS) was used to produce a low resolution envelope of the NisB/NisC/NisA complex allowing to determine the orientation of the individual high resolution crystal structures of NisB and NisC into the SAXS envelope (Figure 7A; Reiners et al., 2017). A comparison of apo-NisB and NisA-saturated NisB suggested the presence of a tunnel and therefore provided an idea for the actual substrate-binding site within the complex. This allowed therefore a first molecular glimpse on the molecular architecture of a maturation complex of a class I lanthipeptide (Figure 7A).

Following the protocol of Ortega et al. (2015) the crystal structure of tRNA^Glu^ (extracted from pdb entry 1N78) was docked into the complex using the HDOCK server^1^ employing standard settings (Yan et al., 2017). The proposed tertiary complex is shown in Figure 7B. Interestingly, the tRNA^Glu^ binding sites are similar in the isolated NisB dimer and the maturation complex. Mapping residues, which result in impaired functionality, cluster around the potential tRNA^Glu^-binding site (Figure 7C) suggesting that the model is of functional significance. Additional residues that were identified in mutational studies were also mapped on the proposed complex (Garg et al., 2013; Khusainov et al., 2013; Ortega et al., 2015). Noteworthy, the mutation of arginine and aspartate residues leading to a complete loss of dehydratase activity in NisB mapped in the close vicinity of the bound tRNA^Glu^ (Figure 7C). Obviously, this *in silico* complex requires experimental verification. Nevertheless, it represents a starting point to design such experiments, which might help to understand the molecular mechanism by which the nisin maturation complex and eventually other maturation complexes of class I lanthipeptides operate.

## Conclusion and Outlook

Over the past years, we have seen tremendous advances in our understanding of lanthipeptides, especially class I lanthipeptides (lantibiotics) on the genetic, functional and structural level. Here, we have focused mainly on the mechanistic and structural insights of the modification process and the corresponding enzymes. The presented findings have answered many questions, but some questions are still open and even new questions arose. Considerably more work will need to be done to understand in detail the molecular coordination and timing of the maturation enzymes and their interplay with the exporter proteins. Only then, the fundamental question of why maturation intermediates of the substrates are not secreted can be answered. Even though literature exhibits great results using lanthipeptide modification enzymes (i.e., synthesis of an analog of angiotensin and an analog of the opioid dermorphin) without full knowledge regarding the mechanisms, a very detailed mechanistic understanding will facilitate higher efficiency regarding drug engineering and design (Luskens et al., 2008; Huo and van der Donk, 2016).

## Author Contributions

All authors wrote the manuscript.

## Conflict of Interest

The authors declare that the research was conducted in the absence of any commercial or financial relationships that could be construed as a potential conflict of interest.

## Footnotes

1 http://hdock.phys.hust.edu.cn
